# Validation of a Remote Sensing Model to Identify *Simulium damnosum* s.l. Breeding Sites in Sub-Saharan Africa

**DOI:** 10.1371/journal.pntd.0002342

**Published:** 2013-07-25

**Authors:** Benjamin G. Jacob, Robert J. Novak, Laurent D. Toe, Moussa Sanfo, Daniel A. Griffith, Thomson L. Lakwo, Peace Habomugisha, Moses N. Katabarwa, Thomas R. Unnasch

**Affiliations:** 1 Global Health Infectious Disease Research Program, Department of Global Health, University of South Florida, Tampa, Florida, United States of America; 2 Multi-disease Surveillance Centre, World Health Organization, Ouagadougou, Burkina Faso; 3 School of Economic, Political and Policy Sciences, University of Texas at Dallas, Richardson, Texas, United States of America; 4 Vector Control Division, Ministry of Health, Kampala, Uganda; 5 The Carter Center, Kampala, Uganda; 6 Emory University and The Carter Center, Atlanta, Georgia, United States of America; University of Queensland, Australia

## Abstract

**Background:**

Recently, most onchocerciasis control programs have begun to focus on elimination. Developing an effective elimination strategy relies upon accurately mapping the extent of endemic foci. In areas of Africa that suffer from a lack of infrastructure and/or political instability, developing such accurate maps has been difficult. Onchocerciasis foci are localized near breeding sites for the black fly vectors of the infection. The goal of this study was to conduct ground validation studies to evaluate the sensitivity and specificity of a remote sensing model developed to predict *S. damnosum* s.l. breeding sites.

**Methodology/Principal Findings:**

Remote sensing images from Togo were analyzed to identify areas containing signature characteristics of *S. damnosum* s.l. breeding habitat. All 30 sites with the spectral signature were found to contain *S. damnosum* larvae, while 0/52 other sites judged as likely to contain larvae were found to contain larvae. The model was then used to predict breeding sites in Northern Uganda. This area is hyper-endemic for onchocerciasis, but political instability had precluded mass distribution of ivermectin until 2009. Ground validation revealed that 23/25 sites with the signature contained *S. damnosum* larvae, while 8/10 sites examined lacking the signature were larvae free. Sites predicted to have larvae contained significantly more larvae than those that lacked the signature.

**Conclusions/Significance:**

This study suggests that a signature extracted from remote sensing images may be used to predict the location of *S. damnosum* s.l. breeding sites with a high degree of accuracy. This method should be of assistance in predicting communities at risk for onchocerciasis in areas of Africa where ground-based epidemiological surveys are difficult to implement.

## Author Summary

Onchocerciasis, or river blindness, represents a major cause of socio-economic disruption throughout much of Africa. The discovery that Mectizan (ivermectin) was effective in treating onchocerciasis, together with the decision of Merck Sharpe and Dohme to donate this drug for the treatment of this disease, revolutionized onchocerciasis control efforts. But until recently, it was thought that ivermectin alone was not sufficient to eliminate this scourge. However, recent studies have suggested that long-term ivermectin treatment can eliminate onchocerciasis in some foci in Africa. This has revolutionized the thinking of the international community, turning its attention from an emphasis on control to an emphasis on elimination. For an elimination program to be successful, it is necessary to accurately map all at-risk communities. In the case of onchocerciasis, this is commonly done by epidemiological surveys. Here we report the validation of a remote sensing method to identify areas endemic for onchocerciasis. This method is based upon predicting the sites where the black fly vector for the parasite breeds. The method was found to be very accurate at predicting black fly breeding sites in West Africa and in northwest Uganda. The method should be useful in assisting in mapping at-risk communities in areas of Africa where ground-based surveys are difficult or impossible to implement.

## Introduction

Onchocerciasis, or river blindness, has historically been one of the most important causes of blindness worldwide [1], [2]. The disease is caused by the filarial parasite *Onchocerca volvulus*. It is estimated that 37 million individuals worldwide are at risk for *O. volvulus* infection, with most residing in rural Africa [3]. In Africa, the parasite is primarily transmitted by black flies of the *Simulium damnosum sensu lato* species complex, which develop as larvae in fast running rivers and streams [3]. Transmission is most intense in and around river basins, rendering many such areas uninhabitable [4]. Unfortunately, areas bordering the river basins contain much of the fertile land found in sub-Saharan African savanna ecosystems. By preventing the agricultural use of the most fertile lands, onchocerciasis has had a significant negative impact on the economic growth of many of the poorest countries of Africa [5].

Treatment and control of onchocerciasis as a public health problem was revolutionized by the discovery that Mectizan (ivermectin) had a potent effect on the larval stages of *O. volvulus* and by the subsequent offer of Merck, Sharpe and Dohme to donate Mectizan free of charge for the treatment of onchocerciasis for as long as needed. This generous donation resulted in the establishment of two major international programs, the African Programme for Onchocerciasis Control, (APOC) and the Onchocerciasis Elimination Program of the Americas (OEPA), whose goals were to control onchocerciasis as a public health problem in Africa (APOC), or to eliminate the parasite from the Americas (OEPA). Both programs utilize community-wide mass distribution of Mectizan as their primary strategy of meeting these goals. OEPA has been successful in interrupting transmission in the majority of onchocerciasis foci in South and Central America [6]. It was initially believed that Mectizan distribution alone could not successfully eliminate onchocerciasis in Africa, due to the widespread distribution of the infection and the intensity of transmission [4]. However, recent data have suggested that this is not the case, and that long-term community wide distribution of Mectizan may be capable of eliminating onchocerciasis in at least some foci in Africa [7]–[10]. This discovery has resulted in a re-focusing of the international community from an emphasis on control of onchocerciasis in Africa towards an emphasis upon possible elimination [11], [12]. However, elimination of onchocerciasis through community-wide Mectizan distribution is logistically difficult, as treatment must be carried out at least annually for many years [13]. Thus, if elimination efforts are to be expanded in Africa, accurate delineation of endemic communities is necessary. Currently, endemic communities are identified through ground-based epidemiological surveys. These can be difficult to conduct in remote and conflict-ridden regions of Africa such as in Southern Sudan and the Democratic Republic of Congo. Thus, methods to identify at-risk communities that cannot be easily reached by ground-based epidemiological surveys are urgently needed.

A common characteristic of all of the members of the *S. damnosum s.l.* species complex is that the immature stages require fast flowing well oxygenated water for development [14]. This means that these flies are quite localized within lotic ecosystems in sub-Saharan Africa. Furthermore, the geographic extent and intensity of human infection is limited by the fact that these insect vectors arise from restricted riverine habitats i.e., fast flowing water. Thus, unlike malaria and many other tropical diseases, the distance that an adult black fly can disperse from its breeding site in search of a blood meal delineates the geographic distribution of onchocerciasis. *Simulium damnosum* s.l. generally travels no further than 12 km from its river source in search of a blood meal [15] and intense transmission of the parasite, resulting in hyper-endemic prevalence levels of disease are generally confined to communities located within 10 km of a river [16], [17]. For these reasons, the common name of “river blindness” has been used to describe onchocerciasis [18]. The ability to identify *S. damnosum s.l.* aquatic larval sites using remote sensing data would be an effective method to delineate areas most at risk for onchocerciasis. The overall goal of this study was to conduct validation studies to evaluate the sensitivity and specificity of a remote sensing model that identifies *S. damnosum s.l.* aquatic larval habitats in Africa.

## Materials and Methods

### 2.1 Study site

Studies in West Africa were conducted along the Sarakawa River in northern Togo. This area has three distinct seasons: warm and dry (November–March), hot and dry (March–May), and hot and wet (June–October). Annual rainfall varies from about 250 mm to 1,000 mm. The terrain is mostly flat with undulating plains and hills. Most of the study site region lies on a savanna plateau, with fields, brush, and scattered trees. The geological history of the site is marked by Precambrian volcanic activity. The study site has four ecological zones or landscapes including: 1) savanna with sparse tree cover; 2) savannas with forested cover, 3) grassy savannas and 4) savannas in temporarily flooded riverine habitats with both forested and grassy adjacent areas. The area is endemic for the savanna-dwelling sibling species of *S. damnosum s.l.* (*S. damnosum s.s.* and *S. sirbanum*) which together represent the two major onchocerciasis vectors in sub-Saharan Africa [18].

### 2.2 Remote sensing models

QuickBird sub-meter satellite data obtained from Digital Globe Inc., (Longmont, CO, USA) were used for this study. The satellite image and data of the study site were acquired on July 15, 2010, roughly at the mid-point of the rainy season. The satellite data contained 25 km^2^ of the land area in the study site. The QuickBird image data were delivered as pan-sharpened composite products in infrared colors. The clearest cloud-free images available of the contiguous sub-areas of the study site along the river and tributaries were used to identify land cover and other spatial features associated with *S. damnosum s.l.* aquatic habitats. The QuickBird imagery was classified using the Iterative Self-Organizing Data Analysis Technique (ISODATA) unsupervised routine in ERDAS Imagine v.8.7 (ERDAS, Inc., Atlanta, Georgia), as previously described [19].

**Figure 1:**
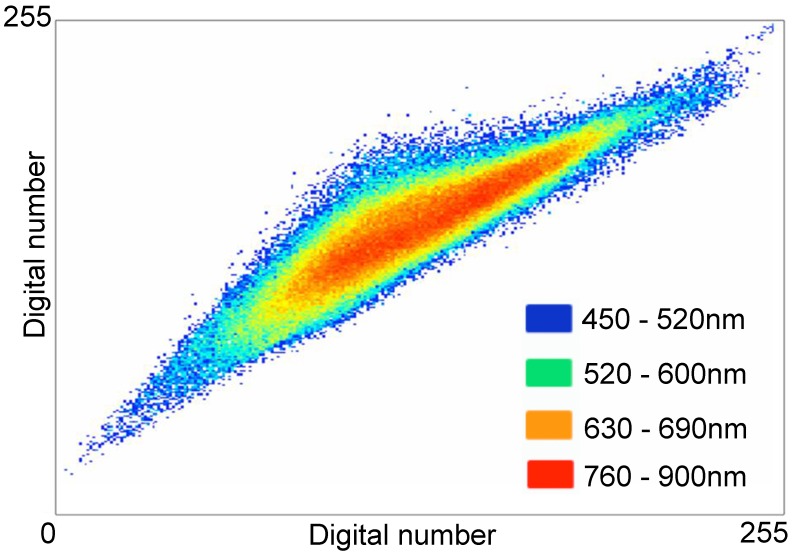
Spectrally decomposed signature of the *S. damnosum* s.l. larval riverine habitat pixel. The signature was extracted from 0.61 m^2^ QuickBird satellite data, as described in the text. The figure depicts the wave band color reflectance ratio (i.e. pixel digital number) of the extracted spectral signature. Colors correspond to the bandwidths indicated in the figure.

The identification of a spectral signature characteristic of *S. damnosum* s.l. positive aquatic sites has been previously described [19]. This spectral signature is characteristic of the habitat features found at known positive sites, which include fast flowing water passing over a substrate of Precambrian rock. The model developed to predict *S. damnosum* s.l. aquatic habitats based on this signature was designated the black rock-rapid (BRR) model. The spectral signature found to be characteristic of the habitat features that formed the basis of the BRR model is shown in Figure 1. The waveband composition data of the signature was 34% red, 11% blue and 55% green.

To develop the BRR model, individual pixel (0.6 m^2^ per pixel) spectral reflectance estimates in the QuickBird images were extracted from georeferenced validated *S. damnosum* s.l. aquatic habitats using a Li-Strahler geometric-optical model, as previously described [20]. This procedure allowed for the creation of a spectral signature of a unit of habitat. The model used three scene components: sunlit canopy (C), sunlit background (G) and shadow (T) generated from the QuickBird image, to determine the sub-pixel endmember spectra associated with the known habitats. The C, G and T component classes were estimated using the ENVI software package (Exelis Visual Information Solutions, Boulder, CO) which employs an object-based classification algorithm [21]. Non-parametric estimators from the endmember spectra and the geometric-optical model were then used to construct a Boolean model that generated a robust spectral signature reference in an ArcGIS database specific for the verified *S. damnosum s.l.* habitats. These unique identifiers of aquatic habitat spectral signatures were then used to predict larval habitats along unsurveyed rivers in both Togo and Uganda. A detailed protocol describing how to utilize the spectral signature to identify *S. damnosum* aquatic habitat is presented supplemental file S1.

To assess the BRR model's ability to predict riverine larval habitat sites that may become temporarily active under varying flow or flooding conditions, a second model was developed. The strategic approach taken was to overlay a Digital Elevation Model with signals characteristic of Precambrian rock plus white water, or Precambrian rock alone. In order to accomplish this, we used PCI Geomatics software (PCI Geomatics, Toronto, Canada), which supported an automatic overlay of an interpolated wet and dry Precambrian rock signature along the river course. This analysis revealed the locations of both active habitats (i.e. those with water flowing over Precambrian rock) as well as sites that might become active under increased flow or flooding conditions. The Digital Elevation Model is a simple tool to locate differences in elevation that would show areas where such fast flowing water could occur during different river flow conditions.

PCI Geomatics Orthoengine software (PCI Geomatics, Toronto, CA) was used to generate a Digital Elevation Model from the QuickBird images. To accomplish this, we used the ArcGIS image server extension supported by version 10.3 of Geomatica Orthoengine for constructing a Toutin's rigorous model, with multiple Rational Polynomial Coefficients (RPC) as previously described [20]. The RPC method used a model developed by Digital Globe, Inc. that approximates a 3-D physical sensor for the QuickBird satellite data. Since bias or error may still have existed in the RPCs representing the interpolated endmember of the signatures, the results were post-processed with a polynomial adjustment product to correct for these biases.

### 2.3 Validation studies

As a first step in assessing the ability of the BRR model to predict the presence of *S. damnosum* s.l. breeding sites, images were collected from the Sarakawa river basin in Northern Togo, which is endemic for the savanna dwelling sibling species of *S. damnosum s.l.* (*S. damnosum sensu strictu* and *S. sirbanum*). These two species together represent the most important onchocerciasis vectors in the savanna regions of sub-Saharan Africa. These images were analyzed to predict potential *S. damnosum s.l.* larval habitats along a 12 km stretch of the river course using the BRR model. The model predictions were then validated by a ground-based validation team, which traversed the 12 km of river by boat. The team included an experienced entomologist employed by the Onchocerciasis Control Program of Burkina Faso. The team visited all of the sites predicted to contain larvae. Sampling was conducted at each site to determine if larvae were present and to estimate the density of the population, if larvae were found. The team also visited and sampled every other site identified by the entomologist as potential *S. damnosum* s.l. aquatic habitat.

**Figure 2:**
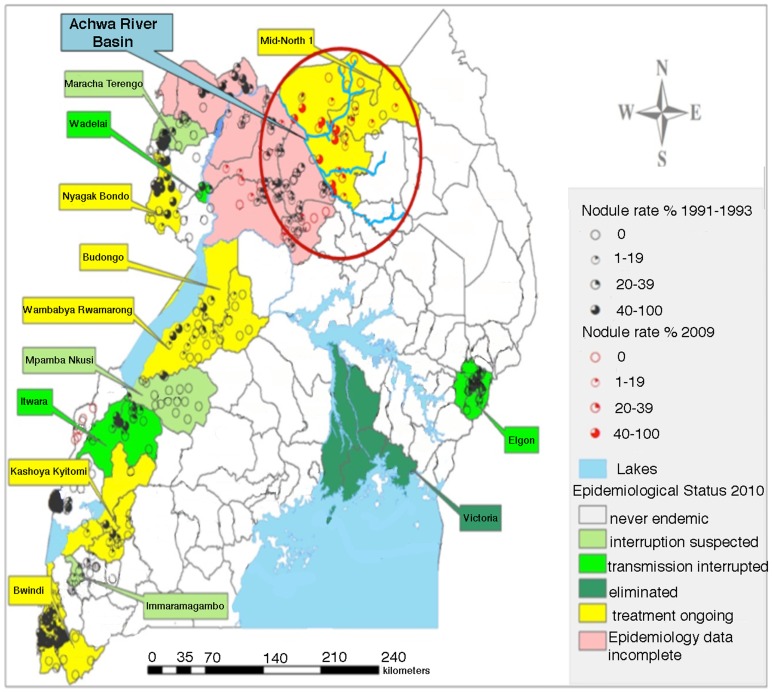
Northern Uganda study site. The map presents data collected by the Ugandan Ministry of Health on the prevalence of onchocerciasis in Uganda. The red circle indicates the location of the Achwa River basin examined in this project.

Validation studies carried out in Northern Uganda along the Achwa River (Figure 2). This area had long suffered from political instability promulgated by the Lord's Resistance Army. As a result, little was known concerning the current prevalence of onchocerciasis in this area. This area presented an ideal opportunity to test the performance of the BRR model in an area where little was known concerning the biology and riverine distribution of black flies and the epidemiology of the disease. The validation studies conducted along the Achwa River employed a similar procedure to that utilized in Northern Togo, with the exception that the sites were visited by walking along the river banks rather than by boating the river course.

## Results

**Figure 3:**
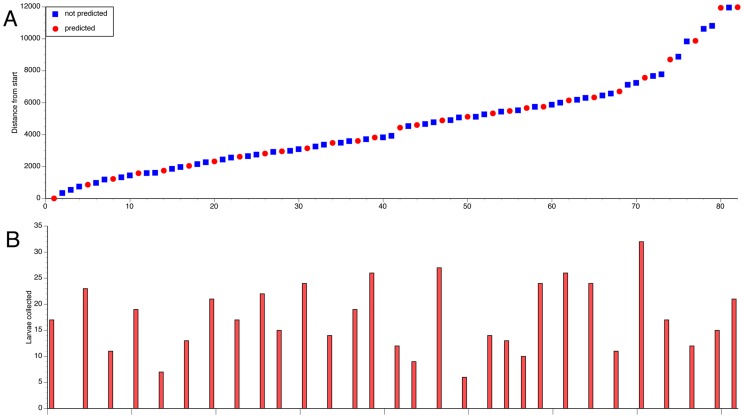
Ground verification of predicted *S. damnosum* s.l. larval habitats. Panel A: Location of sites surveyed. Locations are presented as the Euclidian distance downstream from the start point of the survey (Lat 445861.2662, Lon 1224410.647). Red circles indicate locations predicted to be breeding sites by the model, while blue squares represent locations of other likely larval sites identified by the survey team as described in the text. Panel B: Larval counts from each survey point. Red bars represent larval counts from sites predicted by the model to represent larval habitats and blue bars (all zero) represent larval counts at the other sites surveyed.

Of the 30 sites along the Sarakawa River in Northern Togo predicted to be larval habitats by the BRR model, all (100%) were found to contain *S. damnosum* s.l. larvae. In contrast, none of the 52 sites not predicted by the BRR model, but deemed to be potential habitat by the entomologist accompanying the verification team contained *S. damnosum* s.l. larvae (Figure 3). Together, these data suggested that the BRR model exhibited a sensitivity and specificity approaching 100% for the prediction of *S. damnosum* s.l. riverine larval sites in Togo.

**Figure 4:**
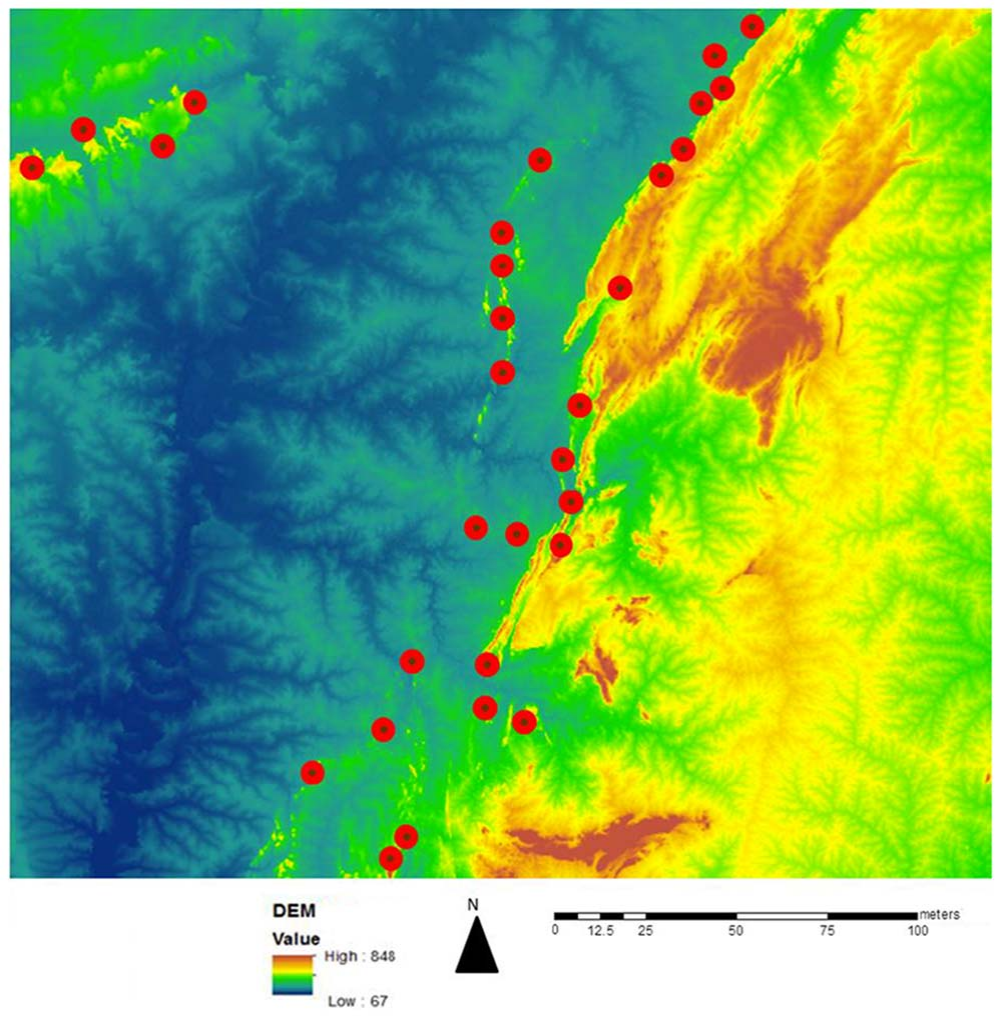
Comparison of habitats predicted by the wet rock and dry rock/elevation change models. Potential *S. damnosum* s.l. breeding habitats were predicted from images collected from the Sarakawa river basin using the BRR model (small black dots) or using a the model based upon the signature for wet or dry Precambrian rock and a sufficient elevation change to support fast flowing water if present (large red dots).

Water levels in the rivers of West Africa fluctuate substantially between the rainy and dry seasons, potentially producing seasonally active breeding sites. A second model was developed to predict such seasonally active sites. There was a complete correspondence between the sites predicted by the the BRR model and this second model (Figure 4), suggesting that the BRR model had identified all active and potentially active breeding sites in the study area.

**Figure 5:**
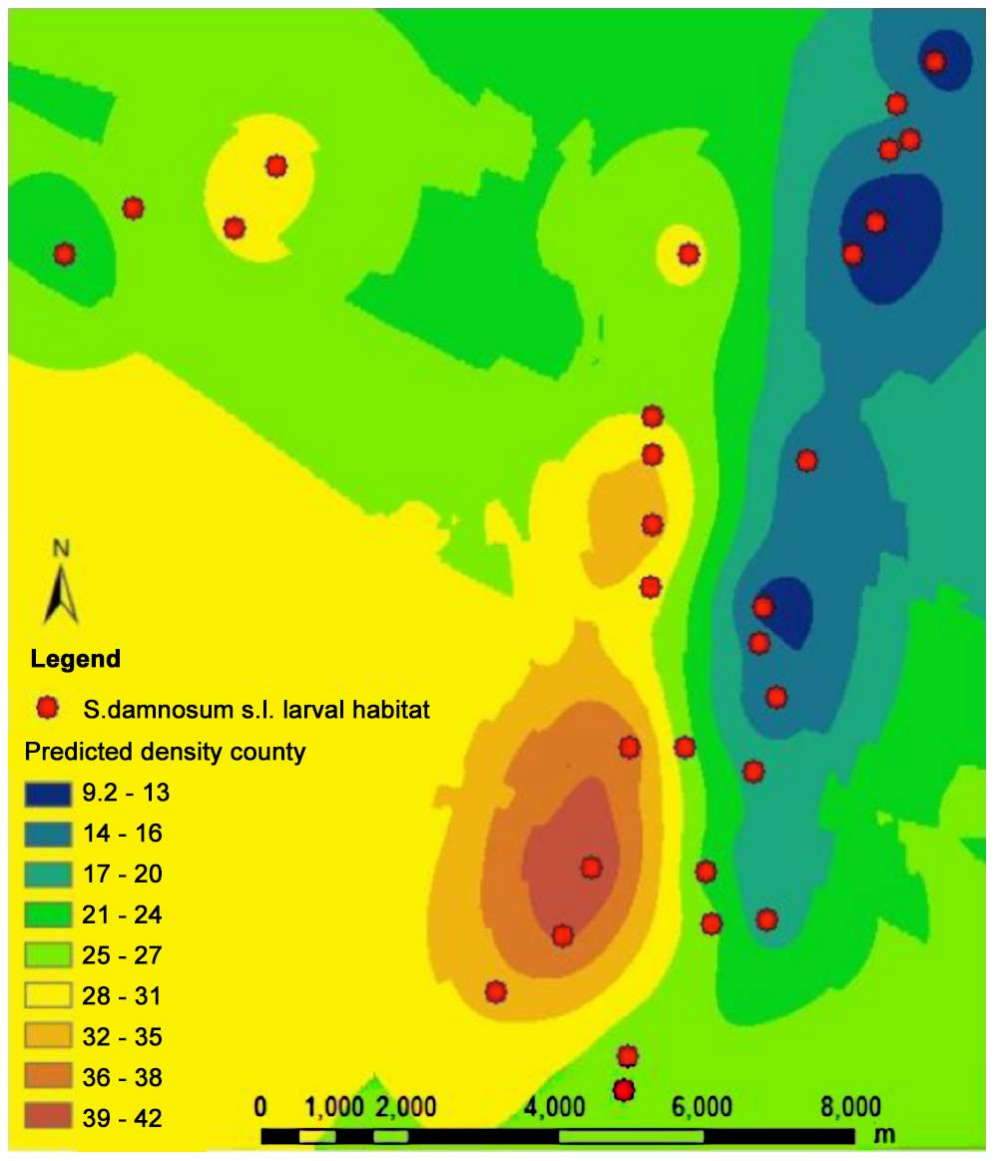
Predicted *S. damnosum* s.l. larval riverine sites in the Achwa River basin. Larval habitats were predicted using the BRR model as described in the text.

**Figure 6:**
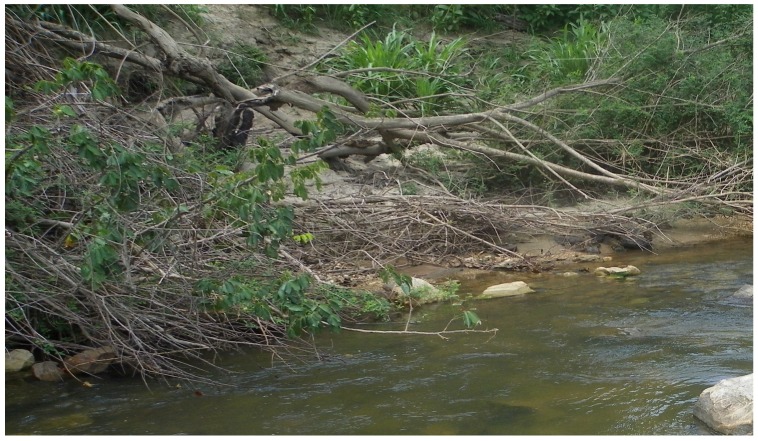
*S. damnosum* s.l. aquatic habitat not predicted by the BRR model. The photo illustrates the hanging vegetation immersed in fast flowing water characteristic of the two breeding sites in the Achwa River not predicted by the BRR model.

*S. damnosum* s.s. and *S. sirbanum* are found in savanna ecosystems throughout most of sub-Saharan Africa. To test the generality of the BRR model, it was applied to predict *S. damnosum* s.l. riverine sites in Northern Uganda. A total of 25 potential *S. damnosum* s.l. larval breeding sites were predicted (Figure 5). Of the 25 sites predicted to be suitable *S. damnosum* s.l. aquatic habitats by the BRR model, 23 (92%; 95% CI 81–100%) were found to contain *S. damnosum* s.l. larvae. In contrast, just 2/10 (20%; 95% CI 0–45%) sites examined which were not predicted to represent *S. damnosum* s.l. aquatic habitat by the model were found to contain larvae. The BRR model thus exhibited a sensitivity of 80% and a specificity of 92% when applied in Uganda, a performance that was statistically significant (p<0.0001; Fisher's Exact test). The two sites that were not predicted by the model which nonetheless were found to contain larvae consisted of low hanging streamside vegetation immersed in fast flowing water (Figure 6). The mean number of larvae found at the sites predicted by the BRR model (21.91) was significantly greater that the mean number of larvae at the sites consisting of immersed overhanging vegetation (4.0; p<0.001, Mann Whitney U test).

## Discussion

It has long been recognized that members of the *Simulium damnosum* s.l. sibling species complex require quite specific ecological conditions for oviposition and for immature development. This species complex requires fast flowing, well oxygenated water in which to develop [14]. This forces the immature stages to localize in areas along rivers and streams where these conditions exist. This key characteristic formed the basis for the vector control activities that underpinned the former Onchocerciasis Control Programme in West Africa (OCP). The OCP's primary strategy was to employ targeted insecticide treatment of *S. damnosum* s.l. aquatic breeding sites to reduce the vector black-fly populations. This strategy was successful in dramatically reducing ocular onchocerciasis in an area encompassing 11 countries [22]. The studies reported here suggest that a remote sensing model based upon the identification of habitat features that are characteristic of these riverine sites was capable of predicting the locations of *S. damnosum* s.l. breeding sites with a high degree of accuracy. The ability to remotely predict the location of *S. damnosum* s.l. breeding sites will be extremely useful in precisely mapping onchocerciasis transmission foci as the current African onchocerciasis control programs move into the era of elimination [11], [12]. In particular, this model may help provide precise mapping of *S. damnosum* s.l. riverine foci in the eastern Democratic Republic of Congo and Southern Sudan, both of which currently suffer from political instability and a lack of infrastructure. Similarly, the ability to predict and precisely locate riverine breeding sites using remote sensing satellite data will provide specific location-based data for mapping the extent of transmission zones in areas that abut international borders, where it is difficult to establish cross border collaborations to conduct ground based studies and coordinated control initiatives. However, while the BRR model may prove to be a useful tool in delineating communities at risk for onchocerciasis and therefore potentially eligible for Mectizan distribution, effectively delivering treatment to such isolated communities will remain a challenge.

The model may also be useful in guiding localized vector control measures that may in some cases could be used to supplement Mectizan distribution. Such measures may need to be considered in areas where biting densities are high [23], [24], or in areas that are co-endemic with the ocular parasite *Loa loa*, as treatment with Mectizan in such co-endemic areas is complicated by the risk of severe adverse events (SAEs) to treatment [25], [26]. The existence of SAEs has limited the use of Mectizan by community directed treatment programs in some areas where onchocerciasis and loasis are co-endemic [27]. The extent of Loasis has previously been mapped using a rapid epidemiologic assessment method (RAPLOA) [28]. It may be possible to employ the BRR model to predict areas likely to be endemic for onchocerciasis in areas previously mapped using the RAPLOA procedure. Combining these maps could identify communities at risk for co-endemicity for both diseases, thereby permitting more complete geographic coverage of the Mectizan community distribution programs while simultaneously minimizing the risk of *L. loa* associated SAEs in the treated areas.

Because there are wide variations in river flow between the wet and dry seasons, it was possible that the BRR model would not detect breeding sites that were seasonally active. However, a test of this hypothesis utilizing a secondary model based solely upon the presence of pre- Cambrian rock (wet or dry) in areas with sufficient elevation change to generate fast flowing water (whether or not water was currently present) did not predict the presence of any additional potentially seasonally active breeding sites. The most likely explanation for this is that the images analyzed by both models were taken in July at the mid-point of the rainy season and all potential breeding sites were active. This suggests that the BRR model may be most accurate when used to analyze images taken during the rainy season. However, because the model predictions were based upon high resolution images (0.6 m^2^) it is also possible that at this degree of resolution the signature characteristic of fast flowing water will be present at all sites during all seasons. Despite this, it is likely that the absolute amount of suitable habitat present at each site will vary according to seasonal changes in water flow. In this regard, it would be of interest to determine if the actual productivity of a breeding site might be predictable based upon the amount of habitat pixels detected by the BRR model. Furthermore, application of the model to images taken at the end of the rainy season or at the beginning of the dry season might be used to predict the most productive breeding sites, permitting a more efficient targeting of localized vector control measures, if such measures become part of the elimination strategic plan in some areas. Studies investigating these possibilities are underway.

The current cost of QuickBird imagery (ca. $17 USD per km^2^) will prove quite expensive if this model is to be applied for predicting *S. damnosum* s.l. breeding habitats on a large scale. However, it may be possible to reduce this cost substantially. First, the number of images necessary might be reduced by limiting the QuickBird image acquisition to river and streambeds, which can be initially identified using free or less expensive data. Alternatively, now that a signature characteristic of *S. damnosum* s.l. larval habitats has been identified with the high-resolution imagery, it may be possible to extract this signature from lower resolution data. It may also be possible to use features extracted from free or less expensive remote sensing data (e.g. vegetation cover, elevation change and river gradient) to pinpoint the regions along a river likely to contain suitable habitats, thereby narrowing down those areas for which QuickBird images need to be obtained. Studies exploring these possibilities are currently underway.

In the validation studies conducted in Uganda, the BRR model did not identify a two breeding sites that were characterized by low hanging riverbank vegetation immersed in fast flowing water. Apparently these sites do not exhibit the spectral signature detected by the BRR model. However, the success of the BRR model suggests that a similar approach might be taken to identify such sites, which would be characterized by immersed vegetation in an area of fast flowing water. Combining this model with the BRR model predictions might be successful in improving the sensitivity of the remote sensing prediction of *S. damnosum* s.l. breeding sites.

The BRR model was developed to predict the riverine locations of the savanna dwelling sibling species of *S. damnosum* s.l., *S. damnosum* s.s. and *S. sirbanum*. While these sibling species represent the most important and most widely distributed onchocerciasis vectors in Africa, the model may not be universally applicable to predict the riverine sites of all *O. volvulus* black fly vectors in Africa. For example, as the current model relies on spectral signatures collected from the visible range, it requires a clear line of sight or relatively large open areas (>5 m) to identify these sites using sub-meter satellite data. Thus, the model will have limited applicability along the coastal rain forested areas of sub-Saharan Africa, where overhanging vegetation often obscures productive riverine sites. Similarly, the current model will not be applicable to the East African vector *Simulium neavei*. *Simulium neavei* has a unique life cycle in which the larvae are phoretic on the bodies of freshwater crabs (*Potamonautes loveni*) found in the streams of Western Kenya and Eastern Uganda [29]. Additional studies will be required to determine if similar remote sensing models might be developed to locate and predict larval breeding habitats areas inhabited by these species.

## Supporting Information

File S1 Protocol for utilizing the Black Rock Rapid Model for predicting *Simulium damnosum* breeding sites.
(DOCX)
