# Supplementary material for: Validation of a Remote Sensing Model to Identify Simulium damnosum s.l. Breeding Sites in Sub-Saharan Africa
Source: PLoS Negl Trop Dis. 2013 Jul 25;7(7):e2342. doi: 10.1371/journal.pntd.0002342 (PMC3723572; doi:10.1371/journal.pntd.0002342)
Supplement: File S1 — Protocol for utilizing the Black Rock Rapid Model for predicting Simulium damnosum breeding sites. (DOCX) [file pntd.0002342.s001.docx]

Protocol for utilizing the Black Rock Rapid Model for predicting *Simulium damnosum* breeding sites

1. Perform a log transformation using the spectrally decomposed 0.61 meter QuickBird *S. damnosum s.l* aquatic habitat endmember data in ArcGIS Spatial Analyst. For *S. damnosum s.l* aquatic habitats the derived BRR must include the waveband ratio of 34% red, 11 % blue and 55% green.
2. Then employ an Ordinary kriged-based model in Spatial Analyst using the QuickBird waveband ratio as the dependent variable in a non-linear regression-based matrix.  Next, to fit the BRR model, employ an exponential empirical semivariogram in Spatial Analyst.  For each bin, form the squared difference from the sampled larval count values for all pairs of habitat locations, and then average and multiply by 0.5 to attain one empirical semivariogram value per bin. Binned points reveal local variation in the semivariogram/covariance values, whereas average values reveal smooth semivariogram/covariance value variation.
3. Binned seasonal-sampled *S. damnosum s.l* aquatic habitats values can then be generated by grouping (binning) empirical semivariogram/covariance points together using square cells that are one lag wide. In Geostatistical Analyst, the lag size and number of lags can then be adjusted to fit. For example, when the larval habitat samples are located on a sampling grid, the grid spacing is usually a good indicator for lag size. A rule of thumb is to multiply the lag size by the number of lags, which should be about half the largest distance among all the sampled larval habitat points. Also, if the range of the fitted semivariogram model is very small relative to the extent of the empirical BRR  model-related semivariogram, the lag size can be decreased. Conversely, if the range of the fitted semivariogram BRR model is large relative to the extent of the empirical semivariogram, the lag size can be increased.
4. To validate the lag size for the BRR interpolator employ the [Average Nearest Neighbor](http://help.arcgis.com/en/arcgisdesktop/10.0/help/005p/005p00000008000000.htm) tool to determine the average distance between the *S. damnosum s.l* aquatic habitats habitat points and there nearest neighbors. The Average Nearest Neighbor tool is located in Spatial Statistics tools under Analyzing Patterns. The Average Nearest Neighbor tool will return five values: Observed Mean Distance, Expected Mean Distance, Nearest Neighbor Index, z-score, and p-value. This would provide a reasonably good lag size for the forecasted *S. damnosum s.l*. riverine habitats, as every lag will have at least a few pairs of geo-referenced larval habitats points in it. The selection of a lag size has important effects on the empirical semivariogram since if the lag size is too large in the residual forecasts the short-range autocorrelation may be masked. Additionally, if the lag size is too small, there maybe many empty bins, and sample sizes within bins will be too small to get representative averages for optimal binning.
5. The empirical semivariogram value in each bin should then be color coded to reveal the optimal BRR model-related semivariogram surface. Then create a semivariogram cloud in Spatial Analyst by plotting half the squared differences of the sampled georeferenced larval habitat points on the y-axis against the distance that separates the geo-referenced habitat points on the x-axis. Within the semivariogram/covariance cloud, spectral characteristics of the interpolated data should be examined for local outliers. An outlying observation, or spatial outlier, is one that appears to deviate markedly from other members of the sample in which it occurs.
6. Once each pair of geo-referenced habitat locations is plotted in Spatial Analyst, the BRR model can be fit into Geostatistical Analyst for estimating the range and the sill. The distance where the model first flattens out is known as the [range](javascript:DictionaryPopup('range')), while the value that the semivariogram model attains at that range (the value on the y-axis) is called the sill
7. The residual forecasts (georeferenced predicted *S. damnosum s.l.* data points) can be input into PROC VARIOGRAM in SAS/GIS, which will compute Moran's *I*  ( a measure of [spatial autocorrelation](http://en.wikipedia.org/wiki/Spatial_autocorrelation)) using binary row standardized and distance weighted  matrices to uncover  the residual dependence in the forecasted data.
8. The final step to constructing a robust seasonal *S. damnosum s.l.* riverine larval habitat endmember predictive model is to generate a validation dataset of random forecasted values for field verification.
